# Safety of Exercise Testing in the Clinical Chinese Population

**DOI:** 10.3389/fcvm.2021.638682

**Published:** 2021-02-09

**Authors:** Yaoshan Dun, Thomas P. Olson, Jeffrey W. Ripley-Gonzalez, Kangling Xie, Wenliang Zhang, Ying Cai, Yuan Liu, Yanan Shen, Nanjiang Zhou, Xun Gong, Suixin Liu

**Affiliations:** ^1^Division of Cardiac Rehabilitation, Department of Physical Medicine and Rehabilitation, Xiangya Hospital of Central South University, Changsha, China; ^2^National Clinical Research Center for Geriatric Disorders, Xiangya Hospital of Central South University, Changsha, China; ^3^Division of Preventive Cardiology, Department of Cardiovascular Medicine, Mayo Clinic, Rochester, MN, United States

**Keywords:** safety, exercise testing, cardiopulmonary exercise testing, cardiovascular disease, clinical Chinese population

## Abstract

This 18-year cross-sectional study was conducted to provide data on the safety of exercise testing in the clinical Chinese population. We retrospectively identified exercise tests completed at Xiangya Hospital of Central South University from January 1, 2002 to December 31, 2019. From 43,130 unique individuals (50.9% female), a total of consecutive 50,142 tests (standard exercise testing 29,466; cardiopulmonary exercise testing 20,696) were retrieved. Demographics, patients' medical history, exercise testing characteristics, and exercise testing-related adverse events were described. Safety data is expressed as the number of adverse events per 10,000 tests, with 95% confidence interval. The average patients' age was 51 ± 13 years. The majority of patients were diagnosed with at least one disease (*N* = 44,941, 89.6%). Tests were maximal or symptom-limited. Common clinical symptoms included dizziness (6,822, 13.6%), chest pain or distress (2,760, 5.5%), and musculoskeletal limitations (2,507, 5.0%). Out of 50,142 tests, three adverse events occurred, including one sustained ventricular tachycardia, one sinus arrest with junctional escape rhythm at a rate of 28 bpm, and one syncopal event with fecal and urinary incontinence. The rate of adverse events was 0.8 events per 10,000 tests (95% confidence interval, 0.2–3.0) in men, 0.4 per 10,000 tests (0.7–2.2) in women, and 0.6 per 10,000 tests (0.21.8) total. This study represents the largest dataset analysis of exercise testing in the clinical Chinese population. Our results demonstrate that clinical exercise testing is safe, and the low rate of adverse events related to exercise testing might be due to the overall changes in clinical practice over time.

## Introduction

Exercise testing, including standard exercise testing (SET) and cardiopulmonary exercise testing (CPX), is commonly used to aid in the diagnosis and prognosis of coronary artery disease (CAD), the assessment of the integrated physiologic function of the cardiovascular, pulmonary, and musculoskeletal systems, and the evaluation of perioperative risk (1–3).

Previous studies (4–7) on the safety of exercise testing from single institutions have been limited to comparatively smaller sample sizes, with the exception of a report by Gibbons et al. (8) This study, preceding the current study by more than 30 years, included 34,295 generally healthy patients (2–4% of patients with a definite medical history) in a preventive medicine clinic and included only SET. Research on larger patient numbers have been conducted *via* large-scale surveys that included results from multiple types of patients from varying facilities with diverse testing protocols (9–12). While these surveys may be convincingly accurate, the authors' assurance that the number of reported tests or complications thereof was complete from the surveyed institutions cannot be provided.

During these intervening years, several studies that have attempted to update and clarify safety issues in exercise testing have been published (13–19). Despite these later publications, limited updates with regards to exercise testing safety have been provided. However, over the past few decades, the utility of exercise testing, particularly CPX, has and continues to expand (1, 2, 20). As such, the increased diversity of patient populations being tested has inevitably increased the complexity of cases and, potentially, the risk of exercise testing. Updated safety data of exercise testing as performed using a standardized procedure in a large sample of patients is warranted.

Importantly, previous studies on the safety of exercise testing predominantly originate from North American and European centers. These data have demonstrated a rate of adverse events ranging from zero to 20.0 per 10,000 tests (4, 5, 8–12, 21, 22). The physiological response, such as exercise capacity (23), to exercise testing may vary by race/ethnicity. Furthermore, there is a paucity of data on the utility and safety of exercise testing in the Chinese population, which limits our understanding of the safety of exercise testing in this large population.

This single-center, 18-year cross-sectional, observational study aims to update our understanding of the safety of exercise testing in the clinical Chinese population.

## Patients and Methods

### Study Design and Patients

This 18-year cross-sectional, observational study retrospectively identified all exercise tests completed at the Cardiac Rehabilitation Center, Xiangya Hospital of Central South University, from January 1, 2002 to December 31, 2019 through an electronic medical record system. Due to patient characteristics such as diagnoses, medication, and exercise function all being subject to change over the 18-year study period, all tests were included for patients who underwent exercise tests multiple times during the study period. A total of 50,142 consecutive exercise tests (SET = 29,466 tests; CPX = 20,696 tests) from 43,130 unique patients were included. The study and application for waiver of written informed consent were reviewed and approved by the Ethics Committee of Xiangya Hospital of Central South University (approval number 202010291). Written informed consent for participation was not provided by either the participants or the participants' legal guardians/next of kin; written informed consent was waived because of the retrospective nature of the study and in accordance with national and institutional requirements. The study protocol conforms to the ethical guidelines of the 1975 Declaration of Helsinki as reflected in *a priori* approval by the institution's human research committee.

### Data Collection

Patients' demographics (sex, age, body weight, height, and body mass index), medical and medication history, adverse events related to exercise testing, and exercise test data were retrieved from an electronic medical record system. All patients were codified and anonymized to protect the confidentiality of individual patients.

### Procedures and Interpretations of Exercise Testing

Both SET and CPX procedures followed the exercise testing guidelines of the American Heart Association (AHA) scientific statement: *Exercise Standards for Testing and Training* (3, 24). Expired gas and/or ECG measurements were conducted using the CARDIOVIT CS-200 (Schiller, Switzerland) for SET and CPX. Exercise modalities included a motor-driven treadmill (Inter-track 8100T, Korea) or cycle ergometry (Ergometer ERG 911 Plus, Germany). Calibration of volume and gas concentration was carried out before the first test of the day, after every significant temperature change, and after changing the sensor. The ambient reference was checked before every calibration. All tests were conducted by a two-person team consisting of a technician with a master's degree in medicine and an exercise professional. All supervisors met the following qualifications: (1) physician license or registered nurse certification, (2) basic life support certification, and (3) more than 4 years of clinical practice experience in the inpatient ward. Each supervisor typically conducted two to four exercise tests per day. Three out of four supervisors had more than 15 years of experience in supervising exercise testing. Additionally, during all exercise testing, a cardiac rehabilitation physician was assigned to the CPX laboratory and was available for questions or concerns.

The SETs were conducted using Bruce and modified Bruce protocols on a treadmill according to the patients' conditions. The CPXs were performed with individually tailored ramp protocols *via* cycle ergometry. The workload for the CPXs was gradually increased each minute by a workload calculated as the value of the predicted maximal work rate divided by 8–12 according to the individual's health condition and activity habit so that the duration of CPX was within 8–12 min (25). A 12-lead ECG was monitored continuously, and blood pressure was measured by manual sphygmomanometry at rest and during the last 20–30 s of every 2 min. Oxygen saturation was monitored continuously *via* finger oximetry (Nellcor N-595 Oximax, Pleasonton, USA). The patients were asked about symptoms and to score their rating of perceived exertion (RPE, Borg 6–20) accordingly at the end of each stage. The patients were encouraged to continue exercising until they presented at least one of the following termination criteria: (1) achievement of ≥85% predicted maximal heart rate (predicted maximal heart rate = 220 - age), (2) plateau in heart rate with increasing workload, (3) plateau in oxygen consumption with increasing workload, (4) respiratory exchange ratio (RER) of ≥1.10, (5) RPE ≥17 (Borg 6–20 scale), (6) angina increasing to three on a scale of four, (7) severe fatigue or dyspnea, (8) a decrease in systolic blood pressure with increasing work rate, (9) severe supraventricular or ventricular arrhythmias, (10) signs of poor peripheral perfusion, or (11) the patient requested to stop (3). A post-test cool-down was performed, with continued ECG monitoring, for 6 min or longer if the patient was symptomatic or if blood pressure, heart rate, and ST segments had not returned to near baseline values. After exercise testing, the patients are asked to stay in the observation area for 30 min. The physician working in the cardiac rehabilitation outpatient clinic would be notified immediately if a patient complains or presents any discomfort that is observed by the on-duty nurse. The physician would evaluate the patient's signs and symptoms and then decide if further medical intervention or emergency department referral is warranted. A medical crash cart, an automatic defibrillator, an inflated oxygen bag, and an ambulance stretcher were available in the observation area.

All test results were first reviewed by the supervising exercise professionals; a cardiologist then conducted a second-round review. Values of peak metabolic equivalents (METs) were reported and further classified as low (<5.0 METs), moderate (5–6.9 METs), high (7–10 METs), and very good (>10 METs) as previously described (26, 27).

### Identification of Adverse Events

Adverse events related to exercise testing were defined as any of the following unexpected events that occurred during or within 24 h after a SET or CPX: (1) death, (2) external defibrillation or implantable cardioverter–defibrillator discharge, (3) sustained supraventricular and ventricular tachycardia (wide complex tachycardia lasting longer than 30 s), (4) myocardial infarction, (5) unstable angina, (6) administration of advanced cardiac life support medications, (7) worsening heart failure, (8) syncope, (9) stroke, (10) transient ischemic attack, (11) referral for direct hospital admission, and (12) direct referral to an emergency department.

All adverse events were identified by reviewing electronic medical records and documented adverse event reports that were routinely created when an adverse event occurred and were stored in the Division of Cardiac Rehabilitation, Xiangya Hospital Central South University. An adverse event report includes a printed completed medical record, physical examination reports, exercise testing report, details of the adverse event, and outcome. To avoid missing data, we re-verified the number of adverse events *via* person-to-person communication with all five faculties who work in the exercise testing laboratory of the Division of Cardiac Rehabilitation, Xiangya Hospital Central South University. Two of them (Suixin Liu, Lihua Deng) are the laboratory initiators (May 2001) and are still working in the laboratory. The rest of them (Lihua Jiang, Qingfang Li) have been working in the laboratory for more than 10 years.

### Sample Size and Power

The Power Analysis & Sample Size software, version 15.0 (NCSS, LLC, USA) was used to calculate power based on our sample. Group sample sizes of sex (men 24,620; women 25,522), exercise type (SET 29,466; CPX 20,696), and medical history (without a diagnosed disease 5,201; with at least one diagnosed disease 44,941) achieve 99.2, 99.2, and 47.5% power, respectively, to detect a difference between the group adverse event rate of 0.001. The adverse event rate in the reference group is assumed to be 0.0001 according to previous studies (5, 8–10). The test statistic used is two-sided Fisher's test. The significance level of the test is 0.05.

### Statistical Analysis

The primary outcome of the present study is the risk of adverse events related to exercise testing in the clinical Chinese population. Safety data was expressed as the number of adverse events per 10,000 tests with a 95% confidence interval. The differences between men and women, SET and CPX, and individuals without a known disease and patients with at least one diagnosed disease in the risk of adverse events related to exercise testing were assessed by Fisher's exact test. The significance level was α = 0.05, two-sided test. Demographics and clinical characteristics of patients were displayed as mean ± standard deviation (*SD*) or number (percent) for continuous and categorical variables accordingly. Analyses were performed using JMP Pro Version 14 (Statistical Analysis System Institute Inc., Cary, NC).

## Results

### Patients' Demographics and Clinical Characteristics

A total of 50,142 exercise tests (50.9% female) were performed from January 1, 2002 to December 31, 2019. Of these, 20,696 (41.3%) were CPX, with the remainder being SET. The average patients' age was 51 ± 13 (mean ± standard deviation) years, and the median (interquartile range) of age was 51 (42–59) years. The vast majority of patients (*N* = 49,628, 99.0%) were adults (age ≥18 years old); among them, 9,727 (19.4%) were older adults (age ≥ 60 years old). The majority of patients presented at least one comorbidity (*N* = 44,941, 89.6%). Among them, 36,659 (73.1%) patients had CVD risk factors (hypertension, dyslipidemia, or diabetes mellitus), 4,972 (9.9%) patients presented established CVD (MI, PCI/CABG), and 2,747 (5.5%) patients presented malignancy (non-small cell lung or esophagogastric cancers). The demographic and clinical characteristics of the patients are described in Table 1. The number of tests by type of test and according to sex and age is provided in Table 2.

**Table 1:** Patients' characteristics.

	**SET (*****N*** **=** **29,466 tests)**		**CPX (*****N*** **=** **20,696 tests)**		**Total (*****N*** **=** **50,142 tests)**	
	**Men**	**Women**	**Men**	**Women**	**Men**	**Women**
	**(*N* = 14,007)**	**(*N* = 15,439)**	**(*N* = 10,613)**	**(*N* =10,083)**	**(*N* = 24,620)**	**(*N* = 25,522)**
Age, years	51 ± 13	51 ± 13	51 ± 13	49 ± 12	51 ± 13	50 ± 13
Body weight, kg	63.7 ± 12.1	55.7 ± 10.0	69.3 ± 11.6	57.7 ± 9.9	66.1 ± 11.9	56.5 ± 10.0
Height, cm	163 ± 8	155 ± 7	167 ± 6	157 ± 6	165 ± 7	156 ± 7
BMI, kg/m^2^	24.0 ± 3.4	23.2 ± 3.5	24.8 ± 3.5	23.4 ± 3.4	23.7 ± 3.4	23.3 ± 3.5
**Exercise modalities**, ***n*** **(%)**						
Treadmill	12,367 (88.3)	13,506 (87.5)	640 (6.0)	386 (3.8)	13,007 (52.8)	13,892 (54.4)
Cycle ergometry	1,640 (11.7)	1,933 (12.5)	9,973 (94.0)	9,697 (96.2)	11,613 (47.2)	11,630 (45.6)
**Medical history**, ***n*** **(%)**^**a**^						
Hypertension	3,754 (26.8)	4,277 (27.7)	4,404 (41.5)	3,640 (36.1)	8,158 (33.1)	7,917 (31.0)
Dyslipidemia	3,488 (24.9)	3,258 (21.1)	2,717 (25.6)	2,319 (23)	6,205 (25.2)	5,577 (21.9)
Diabetes mellitus	2,619 (18.7)	2,671 (17.3)	1,889 (17.8)	1,623 (16.1)	4,508 (18.3)	4,294 (16.8)
Suspected or confirmed CAD	3,698 (26.4)	4,585 (29.7)	2,972 (28.0)	2,470 (24.5)	6,669 (27.1)	7,056 (27.6)
Myocardial infarction	574 (15.5)	540 (11.8)	913 (30.7)	504 (20.4)	1,487 (22.8)	1,045 (14.8)
PCI or/and CABG	546 (14.8)	540 (11.8)	860 (28.9)	494 (20.0)	1,406 (21.1)	1,034 (14.7)
Heart failure	196 (1.4)	262 (1.7)	127 (1.2)	81 (0.8)	323 (1.3)	343 (1.3)
Cardiomyopathy	28 (0.2)	31 (0.2)	96 (0.9)	50 (0.5)	124 (0.5)	81 (0.3)
Congenital heart disease	14 (0.1)	31 (0.2)	21 (0.2)	60 (0.6)	35 (0.1)	91 (0.4)
Valve heart disease	84 (0.6)	108 (0.7)	74 (0.7)	50 (0.5)	158 (0.6)	158 (0.6)
Supraventricular arrhythmia	42 (0.3)	62 (0.4)	605 (5.7)	887 (8.8)	647 (2.6)	949 (3.7)
Cerebrovascular disease	140 (1.0)	77 (0.5)	96 (0.9)	50 (0.5)	236 (1.0)	128 (0.5)
Non-small cell lung cancer	14 (0.1)	31 (0.2)	1,698 (16.0)	928 (9.2)	1712 (7.0)	959 (3.8)
Esophagogastric cancer	2 (0.0)	3 (0.0)	21 (0.2)	50 (0.5)	23 (0.1)	53 (0.2)
Thyroid disease	14 (0.1)	15 (0.1)	32 (0.3)	91 (0.9)	46 (0.2)	106 (0.4)
Other diseases	756 (5.4)	695 (4.5)	499 (4.7)	343 (3.4)	1,255 (5.1)	1,038 (4.1)
Apparently healthy	2,129 (15.2)	1,189 (7.7)	976 (9.2)	907 (9.0)	3,105 (12.6)	2,096 (8.2)
**Medications**, ***n*** **(%)**^**a**^						
Aspirin	1,933 (13.8)	1,914 (12.4)	1,656 (15.6)	1,028 (10.2)	3,589 (14.6)	2,943 (11.5)
Antiplatelet (other than aspirin)	1,177 (8.4)	1,297 (8.4)	1,157 (10.9)	786 (7.8)	2,333 (9.5)	2,083 (8.2)
Anticoagulant	224 (1.6)	293 (1.9)	96 (0.9)	60 (0.6)	320 (1.3)	354 (1.4)
ACEI or ARB	5,085 (36.3)	5,435 (35.2)	4,309 (40.6)	3,297 (32.7)	9,393 (38.2)	8,732 (34.2)
β-Blocker	7,284 (52.0)	83,52 (54.1)	5,720 (53.9)	5,394 (53.5)	13,004 (52.8)	13,747 (53.9)
CCB	4,678 (33.4)	5,496 (35.6)	3,184 (30)	2,682 (26.6)	7,862 (31.9)	8,178 (32.0)
Diuretic	1,177 (8.4)	1,220 (7.9)	711 (6.7)	514 (5.1)	1,888 (7.7)	1,734 (6.8)
Digoxin	266 (1.9)	293 (1.9)	106 (1.0)	40 (0.4)	372 (1.5)	334 (1.3)
Statin	3,362 (24.0)	3,180 (20.6)	4,192 (39.5)	3,368 (33.4)	7,554 (30.7)	6,548 (25.7)
Long-acting nitrate	2,213 (15.8)	2,702 (17.5)	923 (8.7)	615 (6.1)	3,136 (12.7)	3,317 (13.0)
Oral hypoglycemic	2,493 (17.8)	2,470 (16.0)	1,836 (17.3)	1,593 (15.8)	4,329 (17.6)	4,063 (15.9)
Insulin	392 (2.8)	448 (2.9)	159 (1.5)	101 (1.0)	551 (2.2)	549 (2.1)
sNone of these medications	2,409 (17.2)	1,806 (11.7)	1,401 (13.2)	1,311 (13)	3,810 (15.5)	3,117 (12.2)

*SET, standard exercise testing; CPX, cardiopulmonary exercise testing; CAD, Coronary artery disease; PCI, percutaneous coronary intervention; CABG coronary artery bypass surgery; ACEI, angiotensin-converting enzyme inhibitor; ARB, angiotensin II receptor blocker; CCB, calcium channel blocker*.

a *Some patients presented with more than one disease and were prescribed with more than one medication*.

**Table 2:** Number of tests by age, sex, and exercise testing type (*N* = 50,142 tests).

**Age group (year)**	**SET**		**CPX**		**Total**	
	**Men**	**Women**	**Men**	**Women**	**Men**	**Women**
<20	373	290	158	91	531	381
20–29	1,060	1,359	465	638	1,525	1,997
30–39	2,551	2,818	1,199	1,566	3,750	4,384
40–49	4,350	4,162	2,724	2,561	7,074	6,723
50–59	3,413	4,269	3,276	3,092	6,689	7,361
60–69	1,781	2,125	2,035	1,620	3,816	3,745
70–80	436	392	571	375	1,007	767
>80	43	24	185	140	228	164
Total	14,007	15,439	10,613	10,083	24,620	25,522

### Clinical Application of Exercise Testing

**Figure 1:**
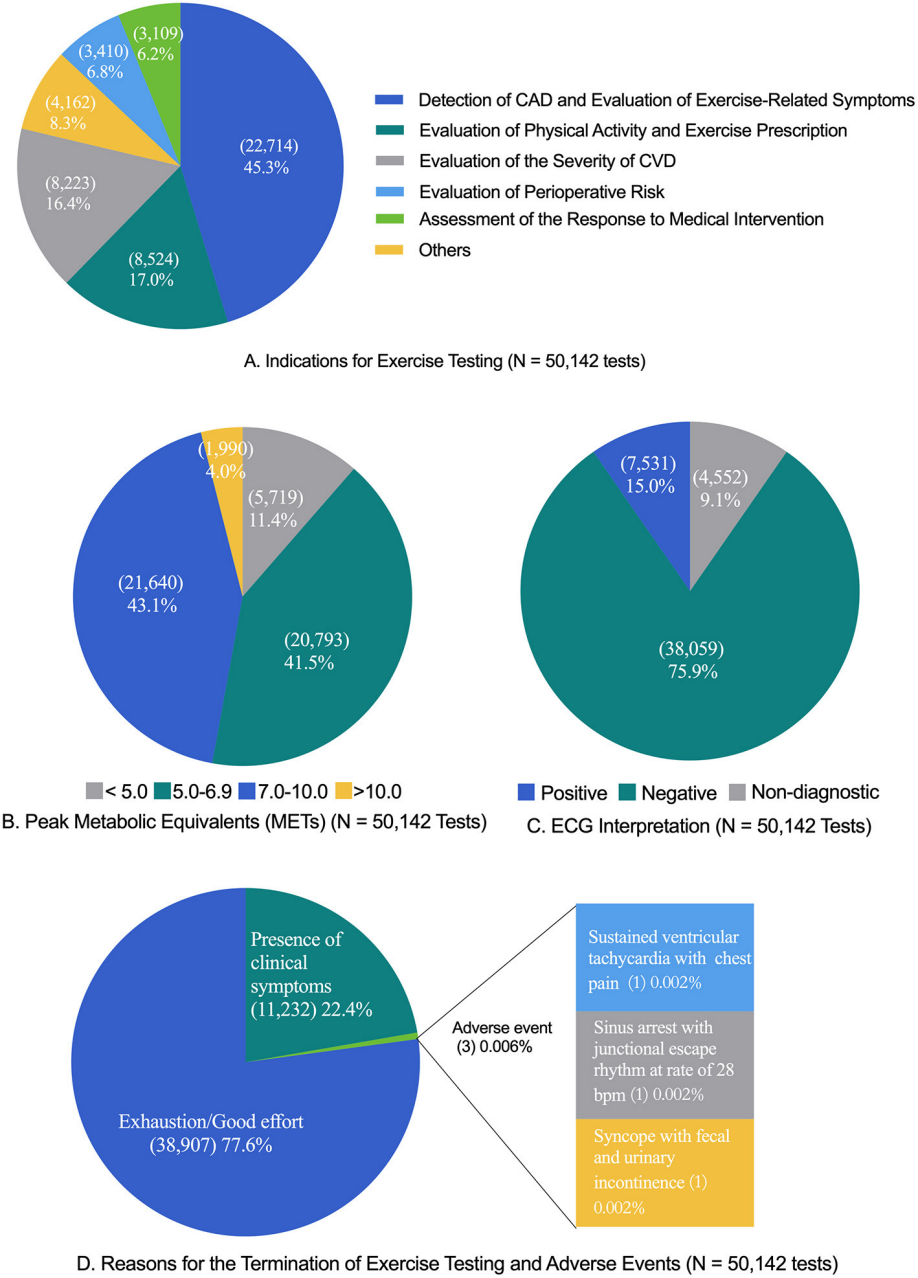
Exercise testing characteristics. CAD, coronary artery disease; CVD, cardiovascular disease; ECG, electrocardiography. The “exhaustion/good effort” criteria included peak heart rate ≥85% of predicted maximal heart rate (220—age) (number, 43,072; percent, 85.9%), the value of respiratory exchange ratio ≥1.10 (6,594, 13.2%; only applicable to cardiopulmonary exercise testing), the score of the rating of perceived exertion ≥17 (44,202, 88.2%), or the presence of the plateau of heart rate or oxygen consumption (401, 0.8%). The common clinical symptoms that resulted in the termination of exercise testing were dizziness (6,822, 13.6%), abnormal blood pressure response (3,796, 7.8%), chest pain or distress (2,760, 5.5%), ST segment elevation or depression ≥3 mm (1,684, 3.4%), and musculoskeletal limit (2,507, 5.0%). The tests were classified as “presence of clinical symptoms” if the patient met the “exhaustion/good effort” criteria and presented a clinical symptom. **(A)** Indications for exercise testing (*N* = 50,142 tests). **(B)** Peak metabolic equivalents (METs) (*N* = 50,142 tests). **(C)** ECG interpretation (*N* = 50,142 tests). **(D)** Reasons for the termination of exercise testing and adverse events (*N* = 50,142 tests).

The majority of patients were referred by a CR physician, cardiologist, or cardiac and thoracic surgeon for at least one proper indication of exercise testing. The data of indications for exercise testing in the patients are provided in Figure 1.

### Results of ECG and Exercise Capacity

The results of exercise ECG and exercise capacity (reported as metabolic equivalents) are shown in Figure 1. For exercise capacity, the mean peak METs were 7.4 ± 1.9 in men and 6.6 ± 1.5 in women for SETs and 7.3 ± 1.6 in men and 6.3 ± 1.3 in women for CPXs.

### Reasons for Exercise Test Termination

The reasons for the termination of exercise testing are shown in Figure 1. The majority of tests were terminated due to the achievement of “good/maximal effort” criteria (38,907; 77.6%), with the remaining tests terminated due to the “presence of clinical symptoms” (11,232; 22.4%).

### Safety of Exercise Testing

Out of the 50,142 tests, three adverse events occurred, two men and one woman (one in SET and two in CPX). The rate of an adverse event in exercise testing was 0.6 per 10,000 tests in total, and no differences were found between men (0.8 per 10,000 tests) and women (0.4 per 10,000 tests) (*P* = 0.62), between SET (0.4 per 10,000 tests) and CPX (0.8 per 10,000 tests) (*P* = 0.57), and between individuals without a diagnosed disease (1.9 per 10,000 tests) and patients with at least one diagnosed disease (0.4 per 10,000 tests) (*P* = 0.28). The 95% confidence intervals for the rate of adverse events were 0.2–3.0 per 10,000 tests in men, 0.7–2.2 per 10,000 tests in women, and 0.2–1.8 per 10,000 tests in total. All three individuals had no known cardiovascular disease but performed pre-tests, such as an ECG and echocardiography, before conducting exercise testing. One patient who was referred for an indication of detecting CAD demonstrated sustained ventricular tachycardia with chest pain during exercise testing, one who was referred for an indication of evaluating perioperative risk experienced sinus arrest with junctional escape rhythm at a rate of 28 bpm in the 1st min of active recovery, and one who was referred for an indication of evaluating perioperative risk experienced syncope with fecal and urinary incontinence at the 10-min mark of sitting recovery. A narrative description of the three adverse events is provided in the Supplementary Appendix, which is summarized in Table 3. A comparison of adverse event rates in previous studies is provided in Table 4.

**Table 3:** Adverse events.

**Patient**	**Indication for exercise testing**	**Pre-test**	**Peak RER**	**Peak HR**	**Exercise capacity**	**Reason for termination**	**Adverse event**	**Management**
62-year-old male with exertional chest discomfort	Detection of CAD	Resting ECG (normal); echocardiography (normal)	NA	160 bpm	<4.6 METs	Sustained ventricular tachycardia with chest pain	Sustained ventricular tachycardia with chest pain	Resolved by administration of sublingual nitroglycerin, supine rest, and oxygen inhalation. Close outpatient follow-up but no hospital admission. Patient has subsequently done well
43-year-old female with mediastinal mass	Evaluation of perioperative risk	Holter ECG (roughly normal); echocardiography (roughly normal)	1.25	173 bpm	20.2 ml/kg/min	Met the termination criteria of fatigue and RER >1.10	Sinus arrest with junctional escape rhythm at rate of 28 bpm	Resolved by supine rest and oxygen inhalation
62-year-old male with right pulmonary nodule	Evaluation of perioperative risk	Holter ECG (suspicious); echocardiography (roughly normal)	1.24	182 bpm	22.2 ml/kg/min	Met the termination criteria of fatigue and RER >1.10	Syncope with fecal and urinary incontinence	Resolved by supine rest and oxygen inhalation

*RER, respiratory exchange ration; HR, heart rate; MET, metabolic equivalent; CAD, coronary artery disease*.

**Table 4:** Exercise testing-related adverse events in other studies.

**Studies (year)**	**Study design**	**Type**	**Main protocol**	**No. of tests/subjects**	**Abnorm. ECG**	**AEs**	**Rate of AEs (per 10,000 tests)**	**Event (no.)**	**Population**	**Location**
Present study	Single-center cross-sectional	SET/CPX	Bruce on treadmill, ramp on cycle	50,142/43,130	Resting (9.1%); exercise (15.0%)	3	0.6	VT (1); sinus arrest (1); syncope (1)	Clinical	China
Kim et al. (4)	Single-center cross-sectional	CPX	Modified Bruce protocol on treadmill	1,485/1,485	Resting and exercise (60.9%)	3	20.0	VTs (3)	Elderly patients with CVD	Korea
Skalski et al. (5)	Single-center, cross-sectional	CPX	Naughton protocols on treadmill	5,060/4,250	Resting (38.0%); exercise (2.5%)	8	16.0	VT (6); AMI (1); severe dyspnea (1)	High-risk CVD	US
Kanthan et al. (6)	Single-center, cross-sectional	SET	Bruce, modified Bruce or Naughton on treadmill	230/230	Exercise (12.2%)	0	NA	NA	Patients who underwent PCI within 7 days	Sydney
Keteyian et al. (7)	Single-center, cross-sectional	CPX	Graded protocol on treadmill	4,411/2,037	Unk	2	4.5	VT (1); VF (1)	Chronic heart failure	US
Gibbons/Cooper et al. (8)	Single-center, cross-sectional	SET	Modified Balke and Ware protocols on treadmill	71,914/34,295	Unk	6	0.8	VT/VF (2); AMI (4)	Generally, only 2–4% of individuals with definite medical history	US
Saito et al. (9)	Survey	SET/ CPX	Treadmill for SET, cycle for CPX	469,215/ (unk)	Unk	41	0.9	3 LAEs; 31 non-LAEs^a^	Clinical	Japan
Myers et al. (10)	Survey	SET^b^	Not reported	32,217/ (unk)	Unk	4	1.2	AMI (3); VT (1)	Clinical	US
Stuart et al. (11)	Survey	ETs	Treadmill, cycle, step test	518,448/ (unk)	Unk	No reported	3.4 AMI, 0.5 deaths, 4.8 arrhythmia	No reported	Clinical	US, Canada, Puerto Rico
Rochmis et al. (12)	Survey	SET	Ramp protocol on treadmill	170,000/ (unk)	Unk	66	4.0	Deaths (16); non-fatal AEs (50)	Clinical	US

*No., number; Abnorm., abnormal; unk, unknown; ET, exercise testing; SET, standard exercise testing; CPX, cardiopulmonary exercise testing; AE, adverse event; VT, sustained ventricular tachycardia; VF, ventricular fibrillation; CVD, cardiovascular disease; AMI, acute myocardial infarction; PCI, percutaneous coronary intervention; Unk, unknown*.

a *Three life-threatening adverse events (LAEs) were acute myocardial infarction; 31 non-life-threatening LAEs included 17 unstable angina, 13 sustained ventricular tachycardias, and one severe orthopedic injury. All AEs occurred during standard exercise testing on treadmill, and none occurred during cardiopulmonary exercise testing on cycle ergometry*.

b *This study also reported nuclear exercise testing and echocardiographic exercise testing but has not discussed these here*.

## Discussion

The present study describes the largest number of patients who completed a SET or CPX done at a single institution ever reported. Moreover, to our knowledge, this is the first study to demonstrate the clinical application and safety of exercise testing, including SET and CPX, in the Chinese population. The tests were performed using the same protocols throughout the entire study period, Bruce or modified Bruce protocols on treadmill and individual tailored ramp protocols through cycle ergometry. All tests were maximal or symptom-limited maximal tests.

The majority of our patients (*N* = 44,941, 89.6%) had at least one known disease at the time of testing, and the adverse event rate was 0.6 per 10,000 tests. This rate is similar to those in the generally healthy individuals (0.8 per 10,000 tests in 71,914 tests) (8). Five other studies in the general patient population have reported adverse event rates ranging from 0.9 to 4.8 per 10,000 tests (7, 9–12). However, adverse event rates vary greatly in the elderly severe/high-risk CVD populations. Kim et al. (4) and Skalski et al. (5) reported that the adverse event rates were 20.0 per 10,000 tests in elderly CVD patients and 16.0 per 10,000 tests in high-risk patients, respectively. A further two studies reported that no adverse events occurred out of 230 tests in CAD patients who performed an early exercise test within 7 days of primary percutaneous coronary intervention (6), and one event of ventricular tachycardia and one of ventricular fibrillation occurred in patients with heart failure out of 4,411 tests (4.5 per 10,000 tests) (7). Our results, in the context of prior studies, suggest that clinical exercise testing is safe across a wide range of patient populations.

The largest surveys on the safety of exercise testing have come from North America, Germany, and Japan, respectively. Rochmis et al. reported an adverse event rate of 4.0 per 10,000 tests from 170,000 exercise tests that were performed in the combined healthy and patient populations in 73 medical centers of the US (12). Wendt et al. reported a rate of 1.1 per 10,000 tests from 1,356,168 tests performed in the general patient population at German clinics (28). Saito et al. reported a rate of 0.9 per 10,000 tests on 469,215 exercise tests performed in patients with CVD or at risk of having CVD in 136 Japanese cardiac rehabilitation centers (9). None of these three survey studies reported further details of the patients' demographic or clinical characteristics.

In previous studies, the most common adverse event was sustained ventricular tachycardia, followed by acute myocardial infarction, across the generally healthy population (8), patient population (7, 9), and elderly patients with severe CVD (4, 5, 7). In these cases, the majority of events were resolved using nasal cannula oxygen therapy, chemical or electrical cardioversions, or cardiopulmonary resuscitation. Our data demonstrate no exercise testing-related death in our facility across a span of 18 years. In contrast, the survey of 518,448 tests in a clinical population by Stuart et al. (11) reported a death rate of 0.5 deaths per 10,000, with a similar rate of 0.9 deaths per 10,000 tests reported by Rochmis et al. (12).

Concerning test safety, a key role may be played by the rate at which exercise intensity increases during the test itself. It has been suggested that the modified Bruce, modified Naughton, and Balke protocols, which use a slower rate of increase in workload, may be safer than the traditional Bruce and Naughton protocols. Currently, the available evidence supports the safety of all protocols; however, insufficient data are available to establish a hierarchy of protocol safety.

Two out of three adverse events in the present study occurred during recovery, with one of these events occurring at 10 min of recovery. Our own experience is consistent with previous studies in that most adverse events occur during recovery rather than during the exercise period of the test. This data suggests that, regardless of whether a patient presents symptoms of or is with or without known CVD, the duration of active cool-down with ECG monitoring should continue for at least 10 min post-exercise or longer if the patient is symptomatic or if the blood pressure, heart rate, and/or ST segments have not returned to near-baseline values.

In our clinical practice, we routinely follow the AHA guidelines (3, 24) regarding absolute and relative contraindications to exercise testing as well as the criteria of exercise termination. With this, our institution includes additional pre-test screening in patients who are at a high risk or who demonstrate overt frailty. The specific components of additional screening vary depending on the patient's condition and are determined by a cardiologist. The most common screening tests include rest ECG, echocardiography, and serum potassium. This practice may be one of the contributors to the lower adverse event rate in the present study than in previously published reports.

Exercise testing across a wide range of patient populations conducted at medical facilities and clinics appears to have become safer over time. A survey of 73 US testing centers in 1971 and a survey of 1,375 US testing centers in 1980 by Rochmis et al. (12) and Stuart et al. (11), respectively, reported overall adverse event rates of 4.0–8.9 events per 10,000 tests. This is in contrast to more recently published event rates in the last two decades (0.6–1.2 events per 10,000 tests) (9, 10). This decrease in the adverse event rate might be due to overall changes in clinical practice over time: improved patient screening, the availability of more skilled personnel, and rapid progress in the field of non-invasive imaging studies, such as computed tomography and nuclear imaging. The patients with a high pre-test probability of CAD or limited exercise capacity might have been referred for computed tomography or nuclear imaging. Those with a lower probability of CAD and with appropriate exercise capacity might have a greater possibility of being referred for exercise testing. This claim is consistent with the data reported. The rate of abnormal ECG, including resting and exercise ECGs, in the present study (24.1%) was lower than those in the two studies (4, 5) reporting higher AE rates (40.5 and 60.9%, respectively) and higher than those in the study (6) that reported a lower AE rate (12.2%) (Table 4).

This study has several limitations. The present study was conducted using single-center data, which may limit the generalizability of the findings, although the large study population with standardized protocols of exercise testing would be a strength of this study. Due to the limit of the retrospective design and only three adverse events that occurred, we are unable to explore the effects of physical activity, exercise capacity, category or severity of diseases, and potential pre-test screening methods on the risk of adverse events and to investigate the temporal changes in adverse event rates. To address these limitations, further prospective multiple-center observational studies are warranted.

## Conclusion

This study demonstrates the safety of exercise stress testing, including SET and CPX, in the Chinese patient population over the past 18 years. This is the largest sample size study to date that examines the safety of exercise stress testing and includes a wide spectrum of disease conditions. The low rate of adverse events related to exercise testing might be due to overall changes in clinical practice over time, such as improved patient screening, more skilled personnel, and rapid progress in the field of non-invasive imaging studies.

## Data Availability Statement

The original contributions presented in the study are included in the article/Supplementary Material, further inquiries can be directed to the corresponding author/s.

## Ethics Statement

The studies involving human participants were reviewed and approved by Ethics Committee of Xiangya Hospital of Central South University. Written informed consent for participation was not provided by the participants' legal guardians/next of kin because: Written informed consent was waived due to the retrospective nature of the study, and in accordance with national and institutional requirements.

## Author Contributions

YD contributed to conceptualization, methodology, writing (original draft), and funding acquisition. TO contributed to conceptualization, writing (review and editing), and visualization. JR-G contributed to writing (original draft), visualization, and formal analysis. KX contributed to resources, validation, and supervision. WZ contributed to investigation, resources, and project administration. YC contributed to resources, data curation, and project administration. YL contributed to resources and project administration. YS contributed to investigation, resources, and project administration. NZ contributed to resources, data curation, and project administration. XG contributed to project administration and data curation. SL contributed to conceptualization, methodology, resources, supervision, and funding acquisition. All authors contributed to the article and approved the submitted version.

## Conflict of Interest

The authors declare that the research was conducted in the absence of any commercial or financial relationships that could be construed as a potential conflict of interest.

## References

[1] Guazzi M Arena R Halle M Piepoli MF Myers J Lavie CJ. 2016 focused update: clinical recommendations for cardiopulmonary exercise testing data assessment in specific patient populations. Circulation. (2016) 133:E694−711. 10.1161/CIR.0000000000000406 27143685

[2] Puente-Maestu L Palange P Casaburi R Laveneziana P Maltais F Neder JA. Use of exercise testing in the evaluation of interventional efficacy: an official ERS statement. Eur Respir J. (2016) 47:429–60. 10.1183/13993003.00745-2015 26797036

[3] Fletcher GF Ades PA Kligfield P Arena R Balady GJ Bittner VA. Exercise standards for testing and training: a scientific statement from the American Heart Association. Circulation. (2013) 128:873–934. 10.1161/CIR.0b013e31829b5b44 23877260

[4] Kim B-J Kim Y Oh J Jang J Kang S-M. Characteristics and safety of cardiopulmonary exercise testing in elderly patients with cardiovascular diseases in Korea. Yonsei Med J. (2019) 60:547–53. 10.3349/ymj.2019.60.6.547 31124338 PMC6536393

[5] Skalski J Allison TG Miller TD The safety of cardiopulmonary exercise testing in a population with high-risk cardiovascular diseases. Circulation. (2012) 126:2465–72. 10.1161/CIRCULATIONAHA.112.110460 23091065

[6] Kanthan A Tan TC Zecchin RP Denniss AR. Early exercise stress testing is safe after primary percutaneous coronary intervention. Eur Heart J Acute Cardiovasc Care. (2012) 1:153–7. 10.1177/2048872612445791 24062903 PMC3760528

[7] Keteyian SJ Isaac D Thadani U Roy BA Bensimhon DR McKelvie R. Safety of symptom-limited cardiopulmonary exercise testing in patients with chronic heart failure due to severe left ventricular systolic dysfunction. Am Heart J. (2009) 158(4 Suppl.):S72–7. 10.1016/j.ahj.2009.07.014 19782792 PMC2762951

[8] Gibbons L Blair SN Kohl HW Cooper K The safety of maximal exercise testing. Circulation. (1989) 80:846–52. 10.1161/01.CIR.80.4.846 2791248

[9] Saito M Ueshima K Saito M Iwasaka T Daida H Kohzuki M. Safety of exercise-based cardiac rehabilitation and exercise testing for cardiac patients in Japan: a nationwide survey. Circ J. (2014) 78:1646–53. 10.1253/circj.CJ-13-1590 24837707

[10] Myers J Voodi L Umann T Froelicher VF. A survey of exercise testing: methods, utilization, interpretation, and safety in the VAHCS. J Cardiopulm Rehabil Prev. (2000) 20:251–8. 10.1097/00008483-200007000-00007 10955267

[11] Stuart RJ Jr. Ellestad MH. National survey of exercise stress testing facilities. Chest. (1980) 77:94–7. 10.1378/chest.77.1.94 7351157

[12] Rochmis P Blackburn H. Exercise tests. A survey of procedures, safety, and litigation experience in ~170,000 tests. JAMA. (1971) 217:1061–6. 10.1001/jama.217.8.1061 5109427

[13] Johnson L Kramer SF Catanzariti G Kaffenberger T Cumming T Bernhardt J. Safety of performing a graded exercise test early after stroke and transient ischemic attack. PM R. (2019) 12:445–53. 10.1002/pmrj.12259 31600415

[14] Tome J Bragg-Gresham J Day S Saberi S Utility and safety of cardiopulmonary exercise testing in hypertrophic cardiomyopathy patients. J Am Coll Cardio. (2018) 71:893 10.1016/S0735-1097(18)31434-7

[15] Scheel PJ Florido R Hsu S Murray B Tichnell C James C. Safety and utility of cardiopulmonary exercise testing in arrhythmogenic right ventricular cardiomyopathy/dysplasia. J Card Fail. (2018) 24:S50–1. 10.1016/j.cardfail.2018.07.146 32009524 PMC7033873

[16] Hashmi S Shaikh S Shaikh A Durrani J Ahmed Z Qutrio Baloch Z Safety of predischarge exercise treadmil test (ETT) after different types of acute myocardial infarction. Eur J Pharm Med Res. (2016) 3:229–33. 10.1136/hrt.2006.106427

[17] Ministeri M Kempny A Montanaro C Alonso-Gonzalez R Swan L Uebing A Safety of cardiopulmonary exercise testing in a large contemporary cohort of adults with congenital heart disease. Eur Heart J. (2016) 37:574–5. 10.1161/JAHA.119.013695

[18] Ha D Mazzone PJ Ries AL Malhotra A Fuster M. The utility of exercise testing in patients with lung cancer. J Thorac Oncol. (2016) 11:1397–410. 10.1016/j.jtho.2016.04.021 27156441 PMC5483326

[19] Ferreira EVM Ramos RP Fonseca ACX Messina CMS Oliveira RKF Costa CM. Safety related to maximal cardiopulmonary exercise testing in patients with pulmonary hypertension. Eur Respir J. (2016) 48:PA2417. 10.1183/13993003.congress-2016.PA241731703589

[20] Radtke T Vogiatzis I Urquhart DS Laveneziana P Casaburi R Hebestreit H. Standardisation of cardiopulmonary exercise testing in chronic lung diseases: summary of key findings from the ERS task force. Eur Respir J. (2019) 54:2019. 10.1183/13993003.01441-2019 31857385

[21] Armstrong MJ Rabi DM Southern DA Nanji A Ghali WA Sigal RJ. Clinical utility of pre-exercise stress testing in people with diabetes. Can J Cardiol. (2019) 35:185–92. 10.1016/j.cjca.2018.11.007 30760425

[22] Komaki K Satomi-Kobayasi S Tsuboi Y Ogawa M Izawa K Sawa T Safety of cardiopulmonary exercise testing for assessing preoperative exercise capacity in Japanese patients with asymptomatic aortic stenosis. J Card Fail. (2015) 21:S174 10.1016/j.cardfail.2015.08.166

[23] Lavie CJ Kuruvanka T Milani RV Prasad A Ventura HO. Exercise capacity in adult African-Americans referred for exercise stress testing: is fitness affected by race? Chest. (2004) 126:1962–8. 10.1378/chest.126.6.1962 15596699

[24] Fletcher GF Balady GJ Amsterdam EA Chaitman B Eckel R Fleg J. Exercise standards for testing and training—A statement for healthcare professionals from the American Heart Association. Circulation. (2001) 104:1694–740. 10.1161/hc3901.095960 11581152

[25] Wasserman K Hansen JE Sue DY Stringer WW Sietsema KE Sun X-G Principles of Exercise Testing and Interpretation-Including Pathophysiology and Clinical Application. 5th ed. Philadelphia, PA: Lippincott Williams & Wilkins (2012). p. 154−78.

[26] Williams MA. Exercise testing in cardiac rehabilitation. Exercise prescription and beyond. Cardiol Clin. (2001) 19:415–31. 10.1016/S0733-8651(05)70226-5 11570114

[27] Peteiro J Bouzas-Mosquera A Broullon F Martinez D Yanez J Castro-Beiras A. Value of an exercise workload >/=10 metabolic equivalents for predicting inducible myocardial ischemia. Circ Cardiovasc Imaging. (2013) 6:899–907. 10.1161/CIRCIMAGING.113.000413 24036386

[28] Wendt T Scherer D Kaltenbach M Life-threatening complications in 1,741,106 ergometries. Dtsch Med Wochenschr. (1984) 109:123–7. 6692777 10.1055/s-2008-1069151

