# Supplementary material for: Safety of Exercise Testing in the Clinical Chinese Population
Source: Front Cardiovasc Med. 2021 Feb 9;8:638682. doi: 10.3389/fcvm.2021.638682 (PMC7900140; doi:10.3389/fcvm.2021.638682)
Supplement: Supplementary file 1 [file Table_1.DOCX]

**Supplementary Appendix**

**Narrative description of three adverse events**

**Patient 1**, a 62-year-old married male, was seen at the cardiology clinic for exertional chest discomfort for three months. He had no known coronary artery disease (CAD), hypertension, diabetes mellitus, and did not use any medication. The patient was able to conduct physical activity at a moderate intensity. Upon physical examination, the resting heart rate was 60 bpm, blood pressure was 122/70 mm Hg. Resting ECG and echocardiography were normal. He was referred for standard exercise testing for the detection of CAD. Bruce exercise testing protocol was performed in the exercise test. At a speed of 1.7 mph and 10% grade on a treadmill, he presented chest pain with ventricular tachycardia at a rate of 160 bpm, a blood pressure of 110/70 mmHg. Supine rest, 0.5-milligram nitroglycerin sublingual tablet, and nasal cannula oxygen therapy were administered. Two minutes later, the ECG turned to sinus rhythm with paroxysmal premature ventricular complexes at a rate of 126 bpm, the chest pain had been alleviated significantly. By 6 minutes of recovery, the ECG was normal, chest pain disappeared. The patient was diagnosed with CAD, and was treated medically, and did well.

**Patient 2**, a 43-year-old married female, was admitted to the thoracic surgery department for the presence of thymomas sized in 33*46 millimeters for one day. She had a history of [myasthenia gravis](https://www.uptodate.com/contents/overview-of-the-treatment-of-myasthenia-gravis?search=Myasthenia+gravis&source=search_result&selectedTitle=1%7E150&usage_type=default&display_rank=1) and hyperthyroidism with standard medication treatment, without known CAD, hypertension, and diabetes mellitus. The physical examination of her heart returned normal, with a resting heart rate of 70 bpm, blood pressure of 150/70 mm Hg. Echocardiography and 24-hour ambulatory (Holter) ECG monitoring were roughly normal. She was referred for exercise testing to evaluate perioperative risk. She completed a cardiopulmonary exercise testing with a cycling ramp protocol in which the work rate increased 12 watts every minute. The peak RPE was 16, RER 1.25, HR 173 bpm, $\dot{V}$O_2_ 20.2 ml/kg/min, without abnormal in the ECG during the testing. The test was terminated for the reason that she had met the termination criteria of fatigue and RER > 1.10. In the first minute of active recovery, she developed a sinus arrest with a junctional escape rhythm at a rate of 28 bpm for 30 seconds, the longest R-R interval was more than two seconds. At the same time, she complained of chest distress, fatigue, and presented facial bloodlessness, cold limbs. Supine rest and nasal cannula oxygen therapy were administered. One minute later, the symptoms had been alleviated, blood pressure was 130/70 mm Hg, HR 68 bpm. She had no further episodes of sinus arrest.

**Patient 3**, a 62-year-old married male, was admitted to the thoracic surgery department for the presence of a right pulmonary nodule sized 12 * 9 millimeters for a half month. He had no known CAD, hypertension, diabetes mellitus, and did not use any medication at the time of their exercise testing. The patient had a sedentary lifestyle and his father had a history of lung cancer. The physical examination of his heart was normal, with a resting heart rate of 71 bpm, blood pressure of 121/77 mm Hg. Holter ECG showed multifocal premature atrial complexes and premature ventricular complexes, accounting for 34‰ and 54‰ of 24-hour total heartbeats respectively, without ventricular tachycardia. Heart rate variability showed a decrease in SDANN index. Echocardiography was roughly normal. He was referred to exercise testing for evaluating perioperative risk. He completed a cardiopulmonary exercise test with the cycling ramp protocol in which the work rate increased 12 watts every minute. At peak work rate, RPE was 16, RER 1.24, HR 182 bpm, $\dot{V}$O_2_ 22.2 ml/kg/min (77 % predicted maximum $\dot{V}$O_2_), without clinically significant change in ECG during exercise. The test was terminated for the reason that he had met the termination criteria of fatigue and RER > 1.10. At ten minutes of sitting recovery, he developed severe syncope with fecal and urinary incontinence. The ECG was reconnected and showed premature ventricular complexes at a rate of 107 bpm, otherwise normal. Blood pressure was 104/74 mm Hg, finger-stick blood glucose was 5.1 mmol/L. Supine rest and nasal cannula oxygen therapy were administered. Within 5 minutes, he recovered consciousness, the HR was 97 bpm，blood pressure 130/90 mm Hg. He was sent back to the thoracic surgery ward. A week later, coronary angiography was performed, with no finding of clinically significant coronary lesions, followed by a right-upper lobectomy without complication.
